# Cluster randomised, controlled, triple-blind trial assessing the efficacy of intranasally administered virus-neutralising bovine colostrum supplement in preventing SARS-CoV-2 infection in household contacts of SARS-CoV-2-positive individuals: a study protocol

**DOI:** 10.1186/s13063-022-06039-9

**Published:** 2022-01-31

**Authors:** Anneli Uusküla, Aime Keis, Karolin Toompere, Anu Planken, Konstantin Rebrov

**Affiliations:** 1 Department of Family Medicine and Public Health, University of Tartu, Ravila 19, 50411 Tartu, Estonia; 2 Icosagen Cell Factory, Eerika tee 1, Kambja vald, 61713 Tartu county, Estonia; 3 Clinic of Oncology, North-Estonian Medical Centre, Sütiste Rd 19, 13419 Tallinn, Estonia; 4 ChemiPharm, Tänassilma road 11, 76406 Tänassilma, Harju county Estonia

## Abstract

**Abstract:**

The SARS-CoV-2 enters into the human body mainly through the nasal epithelial cells. Prevention of SARS-CoV-2 infection at the point of nasal entry is a novel strategy that has the potential to help contain the ongoing pandemic. BioBlock is a nasal spray of anti-SARS-CoV-2 preparation based on virus-neutralising antibodies prepared from colostrum from cows immunised with SARS-CoV-2 spike protein.

This triple-blind placebo-controlled cluster randomised parallel trial seeks to evaluate the efficacy of a BioBlock spray in the prevention and treatment of SARS-CoV-2 infection. Laboratory-confirmed COVID-19 cases and their household members will be randomly allocated to each of either the intervention (BioBlock nasal spray) or the placebo (nasal spray) arms. The intervention is a 14-day course of nasal spray used by index case and household contacts.

In most countries, those with confirmed or suspected infections are requisitioned to isolate at home, putting other members of their household at risk of infection. Therefore, in parallel to the need of household transmission prevention measures, households also present as a good model for infection transmission studies, allowing for the testing of several close contact transmission prevention study hypotheses. Our hope is that if the trial results are encouraging, this will provide new and additional COVID-19 prevention strategies.

**Trial registration:**

ISRCTN48554326 Registered on June 14, 2021

## Administrative information

Note: the numbers in curly brackets in this protocol refer to SPIRIT checklist item numbers. The order of the items has been modified to group similar items (see http://www.equator-network.org/reporting-guidelines/spirit-2727-statement-defining-standard-protocol-items-for-clinical-trials/).

**Table:** 

Title {1}	Cluster Randomised, Controlled, Triple-Blind Trial Assessing the Efficacy of Intranasally Administered Virus-Neutralising Bovine Colostrum Supplement in Preventing SARS-CoV-2 Infection in Household Contacts of SARS-CoV-2 Positive Individuals in Estonia: a study protocol.
Trial registration {2a and 2b}.	This trial is registered at the ISRCTN registry, registration number ISRCTN48554326
Protocol version {3}	Version 2.0 (August 27, 2021).
Funding {4}	ChemiPharm
Author details {5a}	Anneli Uusküla,^a^ Aime Keis,^a^ Karolin Toompere,^a^ Anu Planken,^b,c^ Konstantin Rebrov, ^d^ ^a^ Department of Family Medicine and Public Health, University of Tartu, Ravila 19, Tartu 50411, Estonia^b^ Icosagen Cell Factory, Eerika tee 1, Kambja vald, 61713 Tartu county, Estonia ^c^ Clinic of Oncology, North-Estonian Medical Centre, Sütiste Rd 19, Tallinn, 13419, Estonia ^d^ ChemiPharm, Tänassilma road 11, Tänassilma, 76406 Harju county, Estonia
Coordinating center {5d}	Department of Family Medicine and Public Health, University of Tartu, Estonia
Name and contact information for the trial sponsor {5b}	ChemiPharm Tänassilma road 11, Tänassilma, 76406 Harju county, Estonia
Role of sponsor {5c}	See item 4 (Funding) above.

## Introduction

### Background and rationale {6a}

On 11 March 2020, the World Health Organization (WHO) characterised COVID-19 — a condition caused by the severe acute respiratory syndrome coronavirus 2 (SARS-CoV-2) — as a pandemic. By the mid of 2021, the world is approaching the milestone of four million COVID-19 deaths [1].

Control measures (non-pharmaceutical interventions, including business and school closures, restrictions on movement, total lockdowns, social distancing) have been widely implemented to contain the spread of SARS-CoV-2 and have been effective in curbing the COVID-19 epidemic but do not represent desirable nor sufficient long-term strategies. Multiple vaccines are already at our disposal, but these vaccines will not be sufficient to immunise the majority of the seven billion people on the planet (the volumes needed, costs, cold chain requirements and suboptimal acceptance consist just some of the barriers) [2, 3].

The nose is both a source of pathogens and a critical port of entry for infectious agents such as viruses and bacteria. The SARS-CoV-2 enters into the human body mainly through the nasal epithelial cells [4]. In terms of the current pandemic, some evidence indicates that viral titres of SARS-CoV-2 are extremely high in the nose and throat [5]. The human angiotensin I-converting enzyme 2 (hACE2) receptor, used by SARS-CoV-2 to establish infection, is found to be highly expressed in the mucosa of the oral cavity and nasal tissues [6]. A recent review of nasal sprays and gargles with antiviral properties suggests that a number of commonly used antiseptics might mitigate both the progression and transmission of SARS-CoV-2 [7]. Furthermore, there is evidence from animal experiments that engineered human antibody that recognises SARS-CoV-2 S1 spike administered into mouse nostrils blocks infection in mice that were exposed to high titre SARS-CoV-2 pseudovirus 10 h after the initial antibody treatment. The same experiment indicated that the protection against SARS-CoV-2 infection was effective in both nasal and lung areas 7 days after viral exposure [8]. Other SARS-CoV-2 antibody-based protective nasal sprays are in development. At Stanford University, intranasal administration of antibodies harvested from egg yolks of chickens immunised with spike protein of SARS-CoV-2 is tested for the safety and duration of persistence in the nose [9]. Also, it is a viable research question as to whether the regulation of the systemic inflammatory network against the virus can be modulated, starting from the initial phases of the nasal and nasopharyngeal response [4]. Past studies have shown that airway deliveries of various neutralising anti-influenza monoclonal antibodies (bNAbs) were 10- to 50-fold more effective than systemic deliveries of the same bNAbs in treating H1N1, H3N2 and B/Victoria- and B/Yamagata-lineage influenza viral infections in mouse models [10]. Also, in mice, a single intranasal dose of engineered COVID-19 immunoglobulin M (IgM)-neutralising antibody conferred therapeutic efficacy against SARS-CoV-2 [11].

Since the nose is exposed to different concentrations of environmental agents, it is likely that variable nasal infectivity might influence host immune response and, in turn, the variability of the clinical syndrome of COVID-19 [4]. Prevention of SARS-CoV-2 infection at the point of nasal entry is a novel strategy that has the potential to help contain the ongoing pandemic. Exploring whether directly deploying SARS-CoV-2 antibodies into the upper respiratory airway as a prevention or treatment strategy in an exposed household setting is an obvious research need.

### BioBlock — product development background information

SARS-CoV-2-specific neutralising antibodies can be induced in the blood of cattle by immunisation of pregnant cows with specific viral proteins and the specific antibodies are then excreted to colostrum just before calving. For product development, pregnant cows were immunised three times during the third trimester of pregnancy using SARS-CoV-2 S1 receptor-binding domain (RBD) protein, followed by one boost with SARS-CoV-2 trimeric S protein. The immunoglobulins were purified from colostrum, fat and casein were removed and a safe (pasteurised and filtered) colostrum product was prepared from the protein-rich whey. The ability of the resulting formulation to prevent SARS-CoV-2 virus infection and exhibit antiviral properties has been demonstrated using various in vitro assays, which demonstrated blocking of the interaction between the trimeric S protein and hACE2 [12].

The formulation of purified immunoglobulins was prepared into a nasal spray. A clinical trial to assess the durability of the immunoglobulin nasal spray on nasal mucosal surfaces was conducted on healthy volunteers, which confirmed that the formulation persists on the nasal mucosa for at least 4 h [12, 13].

### Objectives {7}

In attempting to determine the effect of intranasal use of SARS-CoV-2 antiviral antibodies, we planned the BioBlock trial to evaluate the efficacy of a spray in the prevention and treatment of SARS-CoV-2 infection.

1. The main hypothesis of this trial is that using the BioBlock nasal spray by the SARS-CoV-2 carrier and his/her close contacts would be associated with a lower SARS-CoV-2 infection rate in close contact with the SARS-CoV-2 carrier.
2. The secondary hypothesis is that in patients with confirmed SARS-CoV-2 infection, using the BioBlock nasal spray would be associated with improved clinical outcomes.

### Trial design {8}

This is a triple-blind placebo-controlled cluster randomised parallel trial. Clusters, consisting of laboratory-confirmed COVID-19 (index) cases and their household members, will be randomly allocated to each of either the intervention (BioBlock nasal spray) or placebo arms.

A minimum of 124 patients with confirmed COVID-19 (index cases) and their household members will be recruited. Clusters, consisting of an index case and their household members, are randomised (1:1 allocation ratio) to receive application of the BioBlock or to receive placebo spray, both to be administered for 14 days. Participants are contacted on a weekly basis. SARS-CoV-2-infected study subjects are followed up for 21 days to assess symptoms and the course of the disease. Their household members will be tested for SARS-CoV-2 at the baseline and 14-day follow-up and will have exposure information and symptoms recorded. The study will run in two general practitioner (GP) clinics in Tallinn, Estonia.

## Methods: participants, interventions and outcomes

### Study setting {9}

The intervention will be implemented at the general practitioner care level. General practitioners at the two clinics will recruit participants and collect baseline and follow-up data.

### Study population and definitions

The population under investigation consists of the confirmed cases of COVID-19 (irrespective of clinical signs and symptoms) and their close contacts (household members).

For the purpose of this investigation, a household is defined as a group of people living in the same residence. Definition of a household includes two or more people living together in (i) a domestic residence (residential institutions, such as boarding schools, dormitories, hostels or prisons will be excluded) or (ii) a dwelling or group of dwellings with a shared kitchen or common opening onto a shared household space.

Detection of SARS-CoV-2 nucleic acid in a nasopharyngeal specimen will be considered as a confirmed case of COVID-19. COVID-19 cases tested positive within the past 5 days will be considered as newly confirmed [14].

### Eligibility criteria {10}

#### Inclusion and exclusion criteria

Newly laboratory-confirmed cases of COVID-19, aged 16 years or older, with at least one additional household member eligible for participation will be recruited. Household members are eligible for recruitment if they are aged 16 years or older.

Individuals excluded include those who do not live in a household; are pregnant; are receiving active cancer or biological treatment or medications administered by inhalation, or via the naso- and oropharyngeal route; and recovered from confirmed COVID-19 and patients with any organ transplant and with asthma or with known allergies to BioBlock components.

### Who will take informed consent? {26a}

Informed consent will be sought from potential participants by trained research coordinators at study sites. For children aged 16 to 18 years, informed consent to participate will also be sought from their parents.

### Additional consent provisions for collection and use of participant data and biological specimens {26b}

As part of the informed consent process, index patient household members are informed that the study protocol includes the collection of respiratory (nasophryngeal) specimen for SARS-CoV-2 testing and long-term storage of material left over from testing. Samples will be stored in a biobank for potential future studies related to respiratory pathogens, including SARS-CoV-2.

## Interventions

### Explanation for the choice of comparators {6b}

The control condition is placebo, which is ethically justified because no nasally applied agent has definitive proof of efficacy in preventing or treating SARS-CoV-2 infection.

The intervention is a 14-day course of nasal spray used by index case and household contacts.

The experimental group will use the BioBlock intranasally administered virus-neutralising bovine colostrum supplement spray. The spray will be administered to the nasal passages every 4 h.

The control group will use a placebo in the form of a similar spray containing spray base only. The placebo spray will be administered to the nasal passages every 4 h.

Each study subject is allocated one vial of the spray (either active drug or placebo) during the course of the trial.

### Criteria for discontinuing or modifying allocated interventions {11b}

There are no protocol-defined criteria for discontinuing or modifying the study interventions, other than the need for hospitalisation or participant request or, in the case of side effects, requiring of investigation (in all such cases, the use of the study spray will be discontinued).

### Strategies to improve adherence to intervention {11c}

Leaflet with instructions on how to use the spray will be delivered to participants together with the study product. Reading the leaflet will be strongly emphasised in the end of study interview(s).

### Relevant concomitant care permitted or prohibited during the trial {11d}

We have not specified any concomitant care to be permitted or prohibited.

### Provisions for post-trial care {30}

There is no provision for post-trial care in the study, and participants will remain under the care of their usual GP.

### Outcomes {12}

The primary outcome is confirmed SARS-CoV-2 infection in a respiratory specimen (nasopharyngeal swab) by day 14 of the study among the close contacts of a SARS-CoV-2 carrier.

The primary outcome measure is secondary infection rate defined as the frequency of new infections of COVID-19 among contacts of confirmed cases in a defined period of time, as determined by a positive COVID-19 result.

Rate of COVID-19 infection in household members [time frame: 5–14 days]

Acquisition of COVID-19 infection as confirmed by a positive PCR swab taken at the time of symptom onset or at the follow-up visit (day 14)

Secondary outcome measures:

Severity of COVID-19 infection [time frame: 1–21 days]

Time taken for all symptoms to resolve (days)

The number of days until participants report no symptoms, which they attribute to COVID-19, will be compared between groups. Symptoms include fever (38.0 °C or higher); chills; dry cough; rising cough, shortness of breath (rest); shortness of breath (exercise); dyspnoea; sore throat; runny nose; headache; myalgia/bone pain; anorexia; nausea; vomiting; loss of smell; osteoporosis; abdominal pain; diarrhoea; and weakness.

Number of hospital admissions per group [time frame: 1–21 days]

The number of participants admitted to the hospital due to deterioration of their condition due to COVID-19 will be compared between groups.

Number of adverse events per group [time frame: 1–21 days]

The number of adverse events reported will be compared between groups.

Our study timeline is based on the assumptions of a mean incubation period of 5 days and a maximum infectious period of 25 days [15].

*Participant timeline {13}* is presented in Table 1.

**Table 1:** The schedule of enrolment, interventions and assessments

Timepoint	Study period					
	Enrolment	Allocation	Post-allocation			Close-out
	***-t***_***1***_	0	***T***_***1 (7 days)***_	***T***_***2 (14 days)***_	T*_3 (21 days)_	***T***_***x (21 days)***_
Enrolment	x					
Eligibility screen	x					
Informed consent	x					
SARS-CoV-2 RNA testing ^household members^		x				
Allocation		x				
Interventions						
Experimental arm			x	x		
Control arm			x	x		
Assessments						
Sociodemographic characteristics of participants/households; dwelling type and size; household structure; comorbidities; smoking history [16]	x					
Household, occupational and community-related exposures; and utilisation of individual prevention measures [16]	x		x	x	x	
Pharmacovigilance measures			x	x	x	
Outcome variables						
SARS-CoV-2 infection				x^#^		
Severity of COVID-19 infection ^¤^			x	x	x	
Other variables						
Adherence (spray use)			x	x	x	

*Index cases and infected household members only; ^#^or at the time of symptom development; measured by the time taken for all symptoms to resolve (days), and the number of hospital admissions per group

### Sample size {14}

Our target sample size is a minimum of 124 clusters with at least one uninfected household contact at the baseline to be randomised, assuming an average household size of two [17], an intra-class correlation coefficient (*ICC*) of 0.0170 [18], coefficient of variation (of cluster sizes) 0.50 and a secondary infection rate of 30% [19]. This sample size will allow us to detect if the study spray decreases the relative risk for SARS-CoV-2 infection by at least 50%, with 80% power and alpha = 0.05.

### Recruitment {15}

Evidence suggests that the most effective method of recruitment by GPs is opportunistic recruitment [20]. GPs are asked to approach all of their consecutive potentially eligible primary care patients (individuals with a positive SARS-CoV-2 RNA test—“index case”) during the episode of care leading to SARC-CoV-2 testing, check for the first set of inclusion and exclusion criteria and, if appropriate, obtain informed consent for study participation. GPs will review study requirements with the index case and potential eligibility of the household member(s) of the index cases and obtain and document consent. Once the index case has consented to the household study, consent procedures will be initiated for the household members of the index. Members of the household of the index may be recruited into the study during the time of initial contact or an agreed upon alternate time within two business days. Study staff will attempt to approach and recruit all members of the household that the index case has shared contact information. If no household members consent to participate in the study, the index participant will still be enrolled and can continue the study activities. If initially uninfected close contacts of index patients contract SARS-CoV-2 infection (that will be detected either at the day 14 visit or due to symptoms at any point of follow-up), s/he will continue the study under the “index case” protocol (use of the allocated spray for 14 days since infection, and follow-up of the course of the disease at days 7, 14 and 21, after infection detection). All follow-up visits (days 7, 14 and 21) will be phone visits.

The setting for this study is a cluster randomised parallel two-arm RCT, with a cluster (the household consisting of the index patient and his/her household contacts) as a randomisation unit. All cluster members (index and his/her close contacts) will follow the same treatment allocation.

## Assignment of interventions

### Sequence generation {16a}

A randomisation list will be prepared independently by statisticians at the Coordinating Centre using a computer-generated simple randomisation protocol and a 1:1 allocation sequence. A generated randomisation list will be delivered to the investigational product manufacturer (ChemiPharm). Both BioBlock and placebo spray vials have an identical appearance with no treatment allocation displayed. All vials have permanent (non-removable) labels with identifiable serial numbers (Table 2).

**Table 2:** Table to the general practitioner, prepared by the trial statistician

Cluster number	Serial numbers on the vials					Group allocation*
1	TL001	TL001	TL001	TL001	TL001	Placebo
2	TL002	TL002	TL002	TL002	TL002	Placebo
3	TL003	TL003	TL003	TL003	TL003	Active
4	TL004	TL004	TL004	TL004	TL004	Placebo
5	TL005	TL005	TL005	TL005	TL005	Active
6	TL006	TL006	TL006	TL006	TL006	Active
7	TL007	TL007	TL007	TL007	TL007	Active

*Information shielded from the study staff

### Concealment mechanism {16b}

The allocation sequence will be held independently of the research team at GP clinics.

### Implementation {16c}

Once an index participant has completed baseline assessments, the GP will perform the randomisation utilising the centrally held database. Clusters are assigned to a study group sequentially following the randomisation list, and sprays dispensed by the GP. For each cluster to be randomised, five vials of spray are prepared with a unique serial number. GP clinics will use a courier service to deliver assigned investigational products to participating households. The number of vials delivered to a household will depend on the number of consenting study subjects in the household. The vials also have a dedicated place for user name printing (to individualise spray vials).

### Who will be blinded {17a}

The spray vials are blinded at the site of manufacture. The RCT is triple-blind, in which the GP, the study participants and the assessor (at the SARS-CoV-2 RNA testing laboratory) are blinded to the actual spray allocated to the patient. There are no pre-determined situations in which blinding should be broken.

### Procedure for unblinding if needed {17b}

Unblinding will be needed in the event of medical emergencies or serious medical conditions that occur while a participant is taking part in the study whereby the participant may not be able to be treated adequately unless doctors know which treatment they have been receiving. Given the nature of the experimental spray and placebo, we consider this to be an unlikely event. In case of the need for unblinding, the Coordinating Centre needs to be contacted.

## Data collection and management

### Plans for assessment and collection of outcomes {18a}

Assessments will be completed at baseline, after use of the spray (at day 14) and at day 21 (for index cases and infected household members) on follow-up. Data will be collected from the results of an interview and respiratory specimen testing (Table 1).

All interviews will be administered by telephone. Enrolled index cases and SARS-CoV-2 RNA-positive household contacts will complete four phone interviews, including the enrolment interview (day 1) and follow-up interviews on days 7, 14 and 21 of enrolment. Enrolled SARS-CoV-2 RNA-negative household contacts will complete three phone interviews, including the enrolment interview (day 1) and follow-up phone interviews on days 7 and 14.

Data collection questionnaires are based on the WHO household transmission investigation protocol for coronavirus disease 2019 (COVID-19) [16]. We will seek information on the sociodemographic characteristics of participants/households; dwelling type and size; household structure; comorbidities; smoking history; household, occupational and community-related exposures; and utilisation of individual prevention measures. At follow-up, we will assess intervention adherence and occurrence of potential side effects. A visual analogue scale (VAS) for measuring nasal spray use adherence will be used [21]. All responses will be stored in a secure Web platform.

The assessment will include interviews administered remotely via a video-conferencing application or by telephone and Web-based testing referral.

### Specimen collection

Index case COVID-19 confirmatory test results are obtained in routine care at the general practice and the date of testing will be retrieved from participating GPs.

GPs will refer participating household members for SARS-CoV-2 RNA testing to the routine testing sites. In this study, nasopharyngeal swabs will be used for SARS-CoV-2 RNA testing.

Laboratory assessment: The nasopharyngeal samples collected will be tested for SARS-CoV-2 RNA by quantitative reverse-transcriptase–polymerase-chain-reaction (RT-PCR) at the SYNLAB Laboratory, a private medical laboratory company (SolGent DiaPlexQT Novel Coronavirus (2019-nCoV) Detection Kit CE-IVD). Viral RNA from all samples will be isolated within 24 h. The laboratory will provide daily updates to the GP clinics and study Coordinating Centre research staff.

### Plans to promote participant retention and complete follow-up {18b}

Research staff at GP clinics will maximise engagement with participants and facilitate their completion of follow-up assessments. This includes flexibility around the timing of assessments and reminders of missed assessments.

### Data management {19} and confidentiality {27}

Staff at GP clinics will receive training in all aspects of data collection and management. Screening logs and enrolment (identification) logs will be kept at the two GP clinics in locked cabinets within a secured room. All participants (index cases and their household members) will be given a unique study participant identification number. A Web-based electronic data capture system will be designed using REDCAP software. Data will be entered under identification number onto this centrally held database stored on the servers based at the Coordinating Centre. Access to the database will be controlled with unique usernames and encrypted passwords and restricted to members of the research team and external regulators if requested. The servers are protected by firewalls and maintained according to best practice.

The database and associated code lists have been developed by the study coordinators (research team) at the University of Tartu. The database software (REDCAP) provides a number of features to help maintain data quality, including maintaining an audit trail, allowing custom validations on all data, allowing users to raise data-query requests and search facilities to identify validation failure/missing data.

## Statistical methods

We will report participant flow in accordance with the CONSORT principles, with the 2012 extension for reporting cluster trials [22]. Descriptive statistics will be used to summarise assessments of recruitment and completion of the study.

### Statistical methods for primary and secondary outcomes {20a}

Our primary analysis will be a modified intention-to-treat analysis (excluding the clusters with no SARS-CoV-2-negative close contacts at baseline from the analysis). We will randomise clusters (households) prior to knowing the SARS-CoV-2 RNA status of close contacts of index patients (i.e. randomisation will be done before obtaining laboratory information on the SARS-CoV-2 status of household contacts). We argue that given the triple-blinded character of the study, post-randomisation exclusion of clusters with no SARS-CoV-2-negative contacts (exclusion due to excessively broad eligibility criteria [23]) will be random and will not lead into bias [24, 25].

A secondary analysis will be on the intention to treat, including all patients randomised into the study.

BioBlock effectiveness will be estimated using Cox regression (BPE = 1 − hazard ratio [HR]) or Poisson regression (BPE = 1 − rate ratio [RR]). Follow-up will be from baseline to the earliest of outcome or study exit. Cluster robust variance estimator is used to account for clustering.

Both unadjusted and adjusted estimates of BioBlock effectiveness will be presented. Adjustment will be made in the multivariable regression model for main potential confounders (age, symptomatic index cases; household size; sharing bedroom) [26, 27].

Baseline characteristics on family and individual levels will be characterised by descriptive statistics. In case of serious imbalances in variables that are not pre-specified for adjustment, additional sensitivity analysis with additional predictors will be performed.

Number (%) of adverse events and hospitalizations per group are reported. Mean (sd) and median (IQR) duration of symptoms is calculated and compared between groups using linear regression with cluster robust standard errors in case of correlated observations or *t*-test or Wilcoxon rank-sum test.

### Interim analyses {21b}

There are no planned interim analyses.

### Statistical methods to handle missing data {20c}

Every effort will be made to follow up all participants for research assessments. All models will be estimated using maximum likelihood estimation which allows for missing outcome data under the missing-at-random assumption. Sensitivity analyses with worst-case and best-case scenarios are performed.

Statistical analyses will be conducted using Stata software (version 15, StataCorp, College Station, USA). A two-sided *p* value of less than 0.05 will be considered to indicate statistical significance.

### Availability of data and materials {29}

The individualised datasets generated and/or analysed during the current study will not be available. The corresponding statistical code will be available from the research team upon reasonable request, subject to review, following the publication of trial results.

## Oversight and monitoring

### Coordinating Centre {5d}

Research staff at the University of Tartu provide scientific support and oversight and will coordinate all trial activities. The research programme development, design and implementation will be managed by the principal investigator and the co-authors in consultation with GP clinic consultants and experts from the testing laboratory. A dedicated trial coordinator will assist in the day-to-day management of the project at the GP clinics.

### Composition of the data monitoring committee, its role and reporting structure {21a}

As the trial has been assessed to pose a minimal risk to participants, a formal data monitoring committee is not required. The study will be monitored for quality, contextual appropriateness and regulatory compliance. A trial coordinator will monitor the intervention through weekly visits to GP clinics.

### Adverse event reporting and harms {22}

The occurrence of any harms and adverse events will be monitored actively and systematically, following guidance from Consolidated Standards of Reporting Trials (CONSORT). Any adverse event affecting participants on the study observed by GPs or the trial coordinator will be assessed by the principal investigator to decide whether additional investigation, or a modification of the intervention, may be indicated.

### Frequency and plans for auditing trial conduct {23}

The researchers, sponsor and developer will meet regularly (bi-weekly during the initial phase) to monitor progress and conduct of the trial. The main areas of review are (1) ethical review board oversight and consent contents, (2) handling of investigational devices (active and placebo sprays) and (3) patient case review. Auditing visits will be held pre-study (before participant recruitment), after initiation, during the course of study and at close-out.

### Plans for communicating important protocol amendments to relevant parties (e.g. trial participants, ethical committees) {25}

Protocol amendments will be communicated as, and if, required by the principal investigator.

Ancillary and post-trial care {30} will be investigating GP discretion and done according to the Estonian treatment guidelines/clinical routine.

### Dissemination plans {31a}

It is intended that the results of the study will be reported and disseminated at international conferences and in peer-reviewed scientific journals and will be made available to participants and clinical teams in an accessible format and on the study website. The results of the research are expected to be submitted for publishing 6 months after the end of trial data collection. All participants will be fully anonymised in any reporting. In addition, we will produce a short report of findings for each participating GP clinic.

## Discussion

Based on the opinion of more than 100 prominent scientists, coronavirus cannot be eradicated and will become endemic — meaning that it will continue to circulate in pockets of the global population for years to come [28]. A large portfolio of evidence-based prevention and treatment strategies are needed to meet the highly versatile needs of populations.

**Fig. 1:**
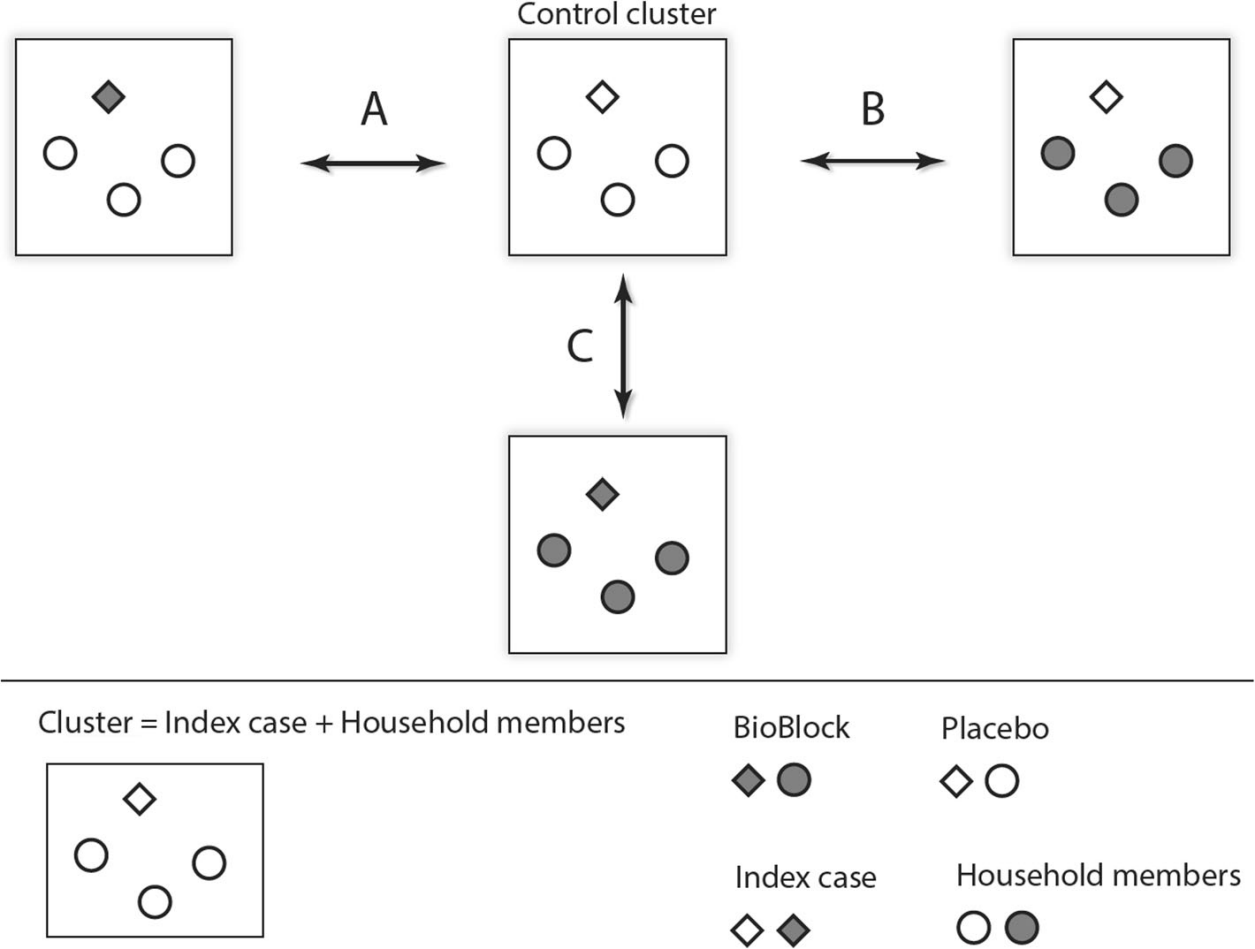
Household-based infection control trials — possible options for using the test device and hypotheses tested. Intervention A, testing the effect on infectiousness: In close contacts of the SARS-CoV-2 carrier, using a study device by the SARS-CoV-2 carrier (only) would be associated with a lower SARS-CoV-2 infection rate among close contacts. Intervention B, testing the effect on susceptibility: In close contacts of the SARS-CoV-2 carrier, using a study device would be associated with a lower SARS-CoV-2 infection rate. Intervention C: In close contacts of the SARS-CoV-2 carrier, using a study device by the close contacts and SARS-CoV-2 carrier would be associated with a lower SARS-CoV-2 infection rate

In most countries, those with confirmed or suspected infections are requisitioned to isolate at home, putting other members of their household at risk of infection. Therefore, in parallel to the need of household transmission prevention measures, households also present as a good model for infection transmission studies, allowing for the testing of several close contact transmission prevention study hypotheses (including infectiousness, susceptibility) (Fig. 1) [29].

The design selected for our study also attempts to give an input for real-life clinical situations where doctors need to treat patients, or implement prevention, measured before all information is available. To be responsive for this type of information need, we have planned an analysis including all randomised clusters (irrespective of whether the cluster includes a participant who would be eligible for infection prevention) as a secondary analysis. One would want to be sure that the benefit of the intervention tested in the study outweighs the harm to patients exposed to the drug without the possibility of benefit. The analysis planned allows interpretation of results both in terms of effectiveness (real-world everyday clinical setting) versus efficacy (resource-intensive ‘ideal setting’) contexts.

The COVID-19 pandemic has led to an unprecedented shift in both health care and research from in-person to remote visits [30]. Our study protocol incorporates several technologies to maintain the safety of trial participants and researchers. First, we use the virtual recruitment (telehealth visits and digitally signed consent forms) and phone interviews for follow-up data collection. Second, Web-based testing referral, and a centrally held database, allows real-time monitoring of the study process. Last, but not the least, the supply of investigational product and study materials will be delivered to participating households via non-contact delivery (courier service).

The results of this BioBlock trial may be generalisable to future trials of primary and secondary COVID-19 prevention and also for testing other than transmission in close contacts. Our hope is that if the trial results are encouraging, this will provide new and additional COVID-19 prevention strategies.

## Trial status

Protocol version 2.0 (August 27, 2021). The recruitment will begin in June 2021 and will be completed by December 31, 2021.

## Data Availability

Data sharing is not applicable to this article as no datasets were generated or analysed during the current study.

## References

[1] ECDC 2021. COVID-19 situation update worldwide, as of week 21, updated 10 June 2021. 2021. https://www.ecdc.europa.eu/en/geographical-distribution-2019-ncov-cases. Accessed 15 June 15 2021

[2] Richman DD (2021). COVID-19 vaccines: implementation, limitations and opportunities. Glob Health Med.

[3] Lazarus JV, Ratzan SC, Palayew A, Gostin LO, Larson HJ, Rabin K, Kimball S, el-Mohandes A (2021). A global survey of potential acceptance of a COVID-19 vaccine. Nat Med.

[4] Gallo O, Locatello LG, Mazzoni A, Novelli L, Annunziato F (2021). The central role of the nasal microenvironment in the transmission, modulation, and clinical progression of SARS-CoV-2 infection. Mucosal Immunol.

[5] Zou L, Ruan F, Huang M, Liang L, Huang H, Hong Z, Yu J, Kang M, Song Y, Xia J, Guo Q, Song T, He J, Yen HL, Peiris M, Wu J (2020). SARS-CoV-2 viral load in upper respiratory specimens of infected patients. N Engl J Med.

[6] Beyerstedt S, Casaro EB, Rangel ÉB (2021). COVID-19: angiotensin-converting enzyme 2 (ACE2) expression and tissue susceptibility to SARS-CoV-2 infection. Eur J Clin Microbiol Infect Dis.

[7] Stathis C, Victoria N, Loomis K, Nguyen SA, Eggers M, Septimus E, Safdar N (2021). Review of the use of nasal and oral antiseptics during a global pandemic. Future Microbiol.

[8] Zhang H, Yang Z, Xiang J, Cui Z, Liu J, Liu C (2020). Intranasal administration of SARS-CoV-2 neutralizing human antibody prevents infection in mice. BioRxiv.

[9] Cohen J. Can a nose-full of chicken antibodies ward off coronavirus infections? Science. 2020. 10.1126/science.abf6581.

[10] Zenilman JM, Fuchs EJ, Hendrix CW, Radebaugh C, Jurao R, Nayak SU, Hamilton RG, McLeod Griffiss J (2015). Phase 1 clinical trials of DAS181, an inhaled sialidase, in healthy adults. Antiviral Res.

[11] Ku Z, Xie X, Hinton PR, Liu X, Ye X, Muruato AE, Ng DC, Biswas S, Zou J, Liu Y, Pandya D, Menachery VD, Rahman S, Cao YA, Deng H, Xiong W, Carlin KB, Liu J, Su H, Haanes EJ, Keyt BA, Zhang N, Carroll SF, Shi PY, An Z (2021). Nasal delivery of an IgM offers broad protection from SARS-CoV-2 variants. Nature.

[12] Kangro K, Kurašin M, Glidemann K, Sankovksi E, Žusinaite E, Lello LS, et al. Bovine colostrum derived antibodies against SARS-CoV-2 show great potential to serve as a prophylactic gent. MedRixV. 2021. 10.1101/2021.06.08.21258069. 10.1371/journal.pone.0268806 PMC9187060 35687549

[13] Clinical trial of BioBlock COVID-19 nasal spray containing anti-SARS-CoV-2 antibodies derived from bovine colostrum. Identifier: NCT04916574. https://clinicaltrials.gov/ct2/show/NCT04916574?cond=NCT04916574&draw=2&rank=1. Accessed 15 June 2021

[14] Byrne AW, McEvoy D, Collins AB, Hunt K, Casey M, Barber A, Butler F, Griffin J, Lane EA, McAloon C, O'Brien K, Wall P, Walsh KA, More SJ (2020). Inferred duration of infectious period of SARS-CoV-2: rapid scoping review and analysis of available evidence for asymptomatic and symptomatic COVID-19 cases. BMJ Open.

[15] Hu B, Guo H, Zhou P, Shi ZL (2021). Characteristics of SARS-CoV-2 and COVID-19. Nat Rev Microbiol.

[16] World Health Organization (2020). Household transmission investigation protocol for coronavirus disease 2019 (COVID-19), version 2.2.

[17] Statistics Estonia. Households. https://andmed.stat.ee/et/stat/sotsiaalelu__leibkonnad Accessed 30 Sept 2021

[18] Picon RV, Carreno I, da Silva AA, Mossmann M, Laste G, Domingues GC, Heringer LFF, Gheno BR, Alvarenga LL, Conte M (2020). Coronavirus disease 2019 population-based prevalence, risk factors, hospitalization, and fatality rates in southern Brazil. Int J Infect Dis.

[19] Madewell ZJ, Yang Y, Longini IM, Halloran ME, Dean NE (2020). Household transmission of SARS-CoV-2: a systematic review and meta-analysis. JAMA Netw Open.

[20] Warren FC, Stych K, Thorogood M, Sharp DJ, Murphy M, Turner KM, Holt TA, Searle A, Bryant S, Huxley C, Taylor RS, Campbell JL, Hillsdon M (2014). Evaluation of different recruitment and randomisation methods in a trial of general practitioner-led interventions to increase physical activity: a randomised controlled feasibility study with factorial design. Trials.

[21] Kalichman SC, Amaral CM, Swetzes C, Jones M, Macy R, Kalichman MO, Cherry C (2009). A simple single-item rating scale to measure medication adherence: further evidence for convergent validity. J Int Assoc Physicians AIDS Care (Chic).

[22] Campbell MK, Piaggio G, Elbourne DR (2012). Altman DG; for the CONSORT Group. Consort 2010 statement: extension to cluster randomised trials. BMJ.

[23] Fergusson D, Aaron SD, Guyatt G, Hébert P (2002). Post-randomisation exclusions: the intention to treat principle and excluding patients from analysis. BMJ.

[24] Exclusions and losses in randomised trials: retention of trial participants. Basicmedicalkey. https://basicmedicalkey.com/exclusions-and-losses-in-randomised-trials-retention-of-trial-participants/. Accessed 15 June 2021

[25] Giraudeau B (2010). Modified intention to treat reporting in randomised controlled trials: systematic review. BMJ.

[26] Li F, Li YY, Liu MJ, Fang LQ, Dean NE, Wong GWK, Yang XB, Longini I, Halloran ME, Wang HJ, Liu PL, Pang YH, Yan YQ, Liu S, Xia W, Lu XX, Liu Q, Yang Y, Xu SQ (2021). Household transmission of SARS-CoV-2 and risk factors for susceptibility and infectivity in Wuhan: a retrospective observational study. Lancet Infect Dis.

[27] Ng OT, Marimuthu K, Koh V, Pang J, Linn KZ, Sun J, de Wang L, Chia WN, Tiu C, Chan M, Ling LM, Vasoo S, Abdad MY, Chia PY, Lee TH, Lin RJ, Sadarangani SP, Chen MIC, Said Z, Kurupatham L, Pung R, Wang LF, Cook AR, Leo YS, Lee VJM (2021). SARS-CoV-2 seroprevalence and transmission risk factors among high-risk close contacts: a retrospective cohort study. Lancet Infect Dis.

[28] Phillips N (2021). The coronavirus is here to stay - here’s what that means. Nature.

[29] Lau LL, Nishiura H, Kelly H, Ip DK, Leung GM, Cowling BJ (2012). Household transmission of 2009 pandemic influenza A(H1N1): a systematic review and meta-analysis. Epidemiology.

[30] Perez T, Perez RL, Roman J (2020). Conducting clinical research in the era of Covid-19. Am J Med Sci.

